# Association between concomitant use of direct oral anticoagulants with antidepressants and an increased risk of hemorrhage: analysis of the food and drug administration adverse event reporting system database

**DOI:** 10.3389/fmed.2026.1765791

**Published:** 2026-02-23

**Authors:** Yuye Ran, Yalan Zhang, Yiwen Cai, Hailin Liu

**Affiliations:** Department of Pharmacy, People’s Hospital of Chongqing Liangjiang New Area, Chongqing, China

**Keywords:** antidepressants, bleeding risk, direct oral anticoagulants, drug interaction, FAERS

## Abstract

**Background:**

Direct Oral Anticoagulants (DOACs) and antidepressants are often co-administered, but their combined effect on bleeding risk in real-world settings is poorly quantified. This study aimed to evaluate this risk using pharmacovigilance data.

**Methods:**

Data spanning quarter 3 (Q3) 2010 to quarter 1 (Q1) 2025 were analyzed from the Food and Drug Administration Adverse Event Reporting System (FAERS) database, comparing bleeding adverse events reported for DOACs monotherapy versus DOACs combined with antidepressants. Bleeding signals were assessed using the Reporting Odds Ratio (ROR).

**Results:**

The proportion of bleeding events was 31.00% (64,165/207,000) in the DOACs monotherapy group versus 57.92% (307/530) in the DOACs-antidepressant combination therapy group. Concomitant use of DOACs with antidepressants was associated with a significant increase in overall bleeding risk (reporting odds ratio [ROR] = 1.45, 95% confidence interval [CI]: 1.29–1.63). Selective serotonin reuptake inhibitors (SSRIs) presented the highest class risk (ROR 1.78, 95% CI 1.54–2.04), with apixaban plus paroxetine showing the strongest signal (ROR 14.12, 95% CI 7.62–26.15). Nervous system bleeding was also elevated (ROR 1.86, 95% CI 1.44–2.40). Notably, mirtazapine significantly increased nervous system bleeding risk (ROR 9.83, 95% CI 4.92–19.67) despite its non-SSRI mechanism.

**Conclusion:**

Co-administration of antidepressants and DOACs significantly elevates bleeding risk, especially for the nervous system. Clinicians must exercise heightened caution, particularly with SSRIs and when using mirtazapine, and further validation studies are needed.

## 1 Introduction

Oral anticoagulants (OACs) are widely prescribed for stroke prevention in patients with atrial fibrillation (AF), prevention and treatment of venous thromboembolism (VTE), and anticoagulant management in patients with heart valves (1–3). While vitamin K antagonists (VKAs), such as warfarin, have served as the cornerstone of anticoagulant therapy for decades, direct oral anticoagulants (DOACs)—have revolutionized clinical anticoagulant practice due to their superior safety profile and simplified medication management (1, 2). Notably, the coexistence of cardiovascular diseases and psychiatric disorders is increasingly prevalent in modern clinical practice, particularly among older adults (4–7), with 4.47–14.1% of patients on oral anticoagulants concomitantly receiving antidepressant therapy (8–10). Selective serotonin reuptake inhibitors (SSRIs), representing a major class of modern antidepressants, are the most widely prescribed in this context (9, 11–13). However, this combination therapy carries a significant risk of bleeding, posing a critical safety concern for clinicians and pharmacists (10, 14–16).

Multiple studies have indicated that antidepressants, especially SSRIs, may independently increase bleeding risk by impairing platelet function (17, 18). When combined with OACs, this risk may be substantially amplified (9, 15, 19). Such synergistic effects can lead to severe adverse events, including gastrointestinal and intracranial hemorrhages, which elevate hospitalization rates and mortality risk, thereby threatening patient safety and necessitating cautious pharmacotherapeutic management (10, 20–22), including careful selection of concomitant medications, dose adjustment, and close monitoring for hemorrhagic events. A 5-year cross-sectional analysis of patients with AF prescribed DOACs revealed that the most common drug classes involved in potential interactions were SSRIs and serotonin-norepinephrine reuptake inhibitors (SNRIs), followed by nonsteroidal anti-inflammatory drugs (NSAIDs), calcium channel blockers (CCBs), and amiodarone. While the overall rate of potential DOAC-related drug interactions slightly declined over the 5-year period, the interaction rate with SSRIs/SNRIs remained relatively stable (23). This underscores the need for heightened vigilance among prescribing clinicians, which should include a comprehensive bleeding risk assessment, review of unnecessary concomitant medications that may increase bleeding risk (such as NSAIDs), consideration of antidepressants with a lower bleeding risk profile when appropriate, use of gastroprotective agents to prevent gastrointestinal bleeding in high-risk patients, closer clinical monitoring for signs of bleeding, and patient education regarding warning symptoms (24–26). These measures can help mitigate the synergistic bleeding risk while maintaining the efficacy of both anticoagulant and antidepressant therapies.

Current evidence on the bleeding risk associated with combined antidepressant and anticoagulant use is limited and has notable constraints. First, well-designed clinical trials often select patients based on strict inclusion and exclusion criteria, which may not reflect real-world patient populations. Second, studies investigating the bleeding risk associated with antidepressants and anticoagulants have primarily focused on SSRIs and SNRIs, leaving uncertainty regarding the impact of other antidepressant classes. Additionally, most studies have employed limited follow-up periods, potentially missing critical events and underestimating the true incidence of adverse events (AEs). Therefore, data from larger-scale, multicenter studies are essential to provide valuable complementary evidence. To address this, we utilized the Food and Drug Administration Adverse Event Reporting System (FAERS) database, the world’s largest real-world AE reporting system for post-marketing surveillance, to aggregate data and identify bleeding signals associated with the co-administration of DOACs and various classes of antidepressants.

## 2 Methods

### 2.1 Data acquisition and processing

Data were obtained from the FAERS database,^1^ covering AE reports associated with medications from the third quarter of 2010 to the first quarter of 2025, and analyzed using R (version 4.3.3). Reports were screened for direct oral anticoagulants (DOACs: rivaroxaban, apixaban, edoxaban, dabigatran) and widely used antidepressants (13, 27–29) (fluoxetine, paroxetine, fluvoxamine, sertraline, citalopram, escitalopram, venlafaxine, desvenlafaxine, duloxetine, bupropion, mirtazapine, agomelatine, trazodone, vortioxetine) based on brand or generic names. Antidepressants were classified by mechanism of action into SSRIs, SNRIs, and other classes (Table 1).

**Table 1:** Classification of antidepressants included in the study

Classification	Antidepressants
SSRIs	Fluoxetine, paroxetine, fluvoxamine, sertraline, citalopram, escitalopram
SNRIs	Venlafaxine, desvenlafaxine, duloxetine
Others	Bupropion, mirtazapine, agomelatine, trazodone, vortioxetine

SSRIs, Selective serotonin reuptake inhibitors; SNRIs, serotonin-norepinephrine reuptake inhibitors.

This table categorizes the 14 antidepressants analyzed in this study by their pharmacological classes, such as SSRIs, SNRIs, and others.

### 2.2 Inclusion and exclusion criteria

Study cases were screened based on all reported medications in the FAERS database, including both primary suspect and concomitant drugs. The non-interaction group included reports where a single DOAC (rivaroxaban, apixaban, edoxaban, or dabigatran) was reported without any other concomitant medications. The interaction group included reports where exactly one DOAC and one of the 14 pre-specified antidepressants were co-administered, with no other concomitant drugs reported. Reports involving multiple anticoagulants, multiple antidepressants, or any additional categories of concomitant medications were strictly excluded to minimize confounding factors from polypharmacy and ensure a precise analysis of the drug–drug interaction.

Data were extracted from the FAERS database, which comprises seven sections: demographics (DEMO), adverse reactions (REAC), patient outcomes (OUTC), drug information (DRUG), therapy start and end dates (THER), report sources (RPSR), and indications/diagnoses (INDI). To eliminate duplicate reports, the FDA-recommended deduplication method was applied, selecting PRIMARYID, CASEID, and FDA_DT fields from the DEMO table. Reports were sorted by CASEID, FDA_DT, and PRIMARYID in that order. For reports with duplicate CASEIDs, the most recent FDA_DT was retained; if multiple reports shared the same CASEID and FDA_DT, the report with the highest PRIMARYID was kept.

### 2.3 Definition of haemorrhagic events

All adverse events were defined using the Medical Dictionary for Regulatory Activities (MedDRA) version 26.1. Hemorrhage reactions were identified using the Standardized MedDRA Query (SMQ code: 20000039). For subgroup analysis of nervous system hemorrhage reactions, events were selected under the High-Level Group Term (HLGT) “Central nervous system vascular disorders.”

### 2.4 Data analysis

Continuous variables in patient demographic data were expressed as means and standard deviations and compared using the *T*-test, with a *p*-value < 0.05 considered statistically significant. Categorical variables were presented as frequencies and proportions, with frequency comparisons conducted using the chi-square test, where a *p*-value < 0.05 indicated statistical significance. Bleeding signals were detected using the reporting odds ratio (ROR), calculated as (*a*/*c*)/(*b*/*d*) based on “interaction/non-interaction” groups (Table 2) (30–32). A bleeding signal was deemed significant when the lower limit of the 95% confidence interval (CI) was >1 and the number of cases in the “interaction” group was ≥3 (Table 2) (32). To adjust for potential confounders (including sex, age, reporting time, and reporter type), signals were further refined using multivariable logistic regression. Logistic regression analyses were performed using R (version 4.3.3), with a two-sided *p*-value <0.05 indicating statistical significance.

**Table 2:** Algorithmic formulae and signal detection criteria.

Method	Formula	Threshold
ROR	$\mathrm{ROR}=\frac{\left(a/b\right)}{\left(c/d\right)}=\frac{\mathrm{ad}}{\mathrm{bc}}$	*a* ≥ 3 and 95% CI (lower limit) > 1
	$\mathrm{SE}(\text{lnROR})=\sqrt{\left(\frac{1}{a}+\frac{1}{b}+\frac{1}{c}+\frac{1}{d}\right)}$	
	$95\%\mathrm{CI}=e^{\ln(\mathrm{ROR})\pm 1.96\sqrt{\left(\frac{1}{a}+\frac{1}{b}+\frac{1}{c}+\frac{1}{d}\right)}}$	

In the ROR calculation, variables are defined as follows: *a* represents the number of bleeding AEs in the interaction group (DOACs combined with antidepressants), *b* represents the number of non-bleeding AEs in the interaction group, *c* represents the number of bleeding AEs in the non-interaction group (DOAC monotherapy), and *d* represents the number of non-bleeding AEs in the non-interaction group.

Details the mathematical equations for ROR used in this study, along with the specific thresholds required to define a positive safety signal.

## 3 Results

### 3.1 Demographics and patient characteristics

**Figure 1:**
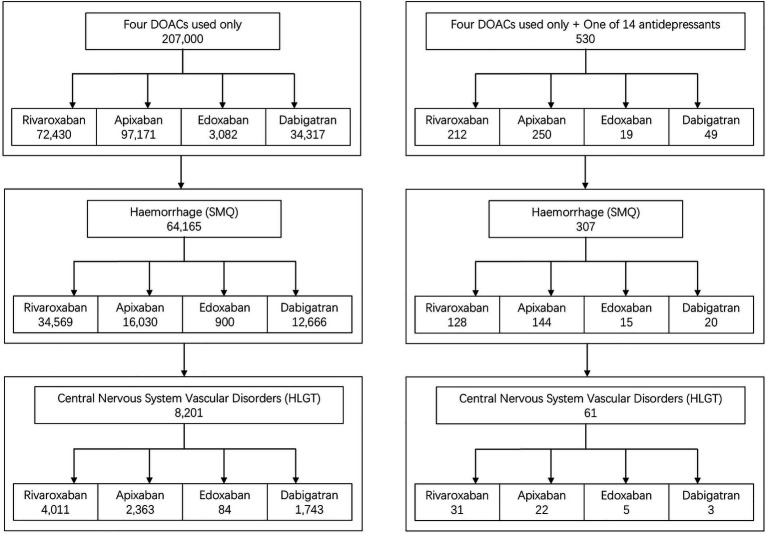
Study flowchart. Illustrates the stepwise screening process from the total FAERS database to the final 530 cases in the interaction group and 207,000 cases in the DOAC monotherapy group.

Figure 1 illustrates the study flowchart. From the third quarter of 2010 to the first quarter of 2025, a total of 207,000 AEs associated with DOAC monotherapy were identified, including 64,165 bleeding events (31.0%). The number and proportion of bleeding events for each DOAC were as follows: rivaroxaban, 34,569 cases (47.73%); apixaban, 16,030 cases (16.50%); edoxaban, 900 cases (29.20%); and dabigatran, 12,666 cases (36.91%). Among bleeding events, nervous system bleeding cases and their respective proportions were: rivaroxaban, 4,011 cases (11.60%); apixaban, 2,363 cases (14.74%); edoxaban, 84 cases (9.33%); and dabigatran, 1,743 cases (13.76%).

A total of 530 AEs were identified for DOACs co-administered with any of the 14 antidepressants (without other concomitant medications), including 307 bleeding events (57.92%) and 410 preferred terms (PTs). The number and proportion of bleeding events for each DOAC combined with antidepressants were: rivaroxaban, 128 cases (60.38%); apixaban, 144 cases (57.60%); edoxaban, 15 cases (78.95%); and dabigatran, 20 cases (40.82%). Among these bleeding events, nervous system bleeding cases and their respective proportions were: rivaroxaban, 31 cases (24.21%); apixaban, 22 cases (15.28%); edoxaban, 5 cases (33.33%); and dabigatran, 3 cases (15.0%).

Table 3 presents the baseline characteristics of patients in the interaction and non-interaction groups, showing significant differences in reporting age, sex, date, and reporter type (*p* < 0.001). The interaction group had a higher median age (78 vs. 74 years) and a greater proportion of females (59.2% vs. 51.0%). Physicians were the most frequent reporters in the interaction group (34.5%), while consumers were the primary reporters in the non-interaction group (55.2%). In terms of geographical distribution, the top three regions were the United States, Germany, and Japan, with 156,230, 7,685, and 7,101 cases reported, respectively.

**Table 3:** Baseline characteristics of patients, this table compares the demographic data and reporter profiles among the overall population, the interaction group (concomitant use of a single DOAC and a single antidepressant), and the non-interaction group (DOAC monotherapy).

Characteristic	Overall *N* = 207,530	Interaction *N* = 530	Non-interaction *N* = 207,000	*p*-value
Age (years), median (Q1, Q3)	74 (63, 82)	78 (63, 86)	74 (63, 82)	<0.001
Age (years)				<0.001
0–17	4,177 (3.9%)	5 (1.3%)	4,172 (3.9%)	
18–59	16,794 (15.6%)	81 (21.5%)	16,713 (15.6%)	
60–74	33,666 (31.3%)	73 (19.4%)	33,593 (31.4%)	
≥75	52,850 (49.2%)	218 (57.8%)	52,632 (49.1%)	
Missing	100,043	153	99,890	
Sex				<0.001
Female	89,814 (51.1%)	284 (59.2%)	89,530 (51.0%)	
Male	86,071 (48.9%)	196 (40.8%)	85,875 (49.0%)	
Missing	31,645	50	31,595	
Report date				<0.001
2010	216 (0.1%)	0 (0%)	216 (0.1%)	
2011	6,295 (3%)	4 (0.8%)	6,291 (3%)	
2012	6,802 (3.3%)	4 (0.8%)	6,798 (3.3%)	
2013	7,551 (3.6%)	10 (1.9%)	7,541 (3.6%)	
2014	9,139 (4.4%)	12 (2.3%)	9,127 (4.4%)	
2015	18,013 (8.7%)	36 (6.8%)	17,977 (8.7%)	
2016	17,932 (8.6%)	30 (5.7%)	17,902 (8.6%)	
2017	18,868 (9.1%)	26 (4.9%)	18,842 (9.1%)	
2018	18,060 (8.7%)	60 (11.3%)	18,000 (8.7%)	
2019	20,203 (9.7%)	74 (14%)	20,129 (9.7%)	
2020	22,254 (10.7%)	35 (6.6%)	22,219 (10.7%)	
2021	19,774 (9.5%)	23 (4.3%)	19,751 (9.5%)	
2022	16,362 (7.9%)	86 (16.2%)	16,276 (7.9%)	
2023	11,929 (5.7%)	48 (9.1%)	11,881 (5.7%)	
2024	11,913 (5.7%)	74 (14%)	11,839 (5.7%)	
2025	2,219 (1.1%)	8 (1.5%)	2,211 (1.1%)	
Reporter type				<0.001
Physician	56,753 (27.5%)	182 (34.5%)	56,571 (27.5%)	
Pharmacist	13,972 (6.8%)	76 (14.4%)	13,896 (6.7%)	
Lawyer	402 (0.2%)	0 (0%)	402 (0.2%)	
Consumer	113,753 (55.1%)	176 (33.3%)	113,577 (55.2%)	
Other health professional	21,521 (10.4%)	94 (17.8%)	21,427 (10.4%)	
Missing	1,129	2	1,127	
Top 3 reported country (*n*)	United States (156,230)	United States (190)	United States (156,040)	–
	Germany (7,685)	France (162)	Germany (7,651)	
	Japan (7,101)	Spain (45)	Japan (7,100)	

Missing: denotes records where the information was not recorded by the reporter in the FAERS database.

### 3.2 Bleeding risk

**Figure 2:**
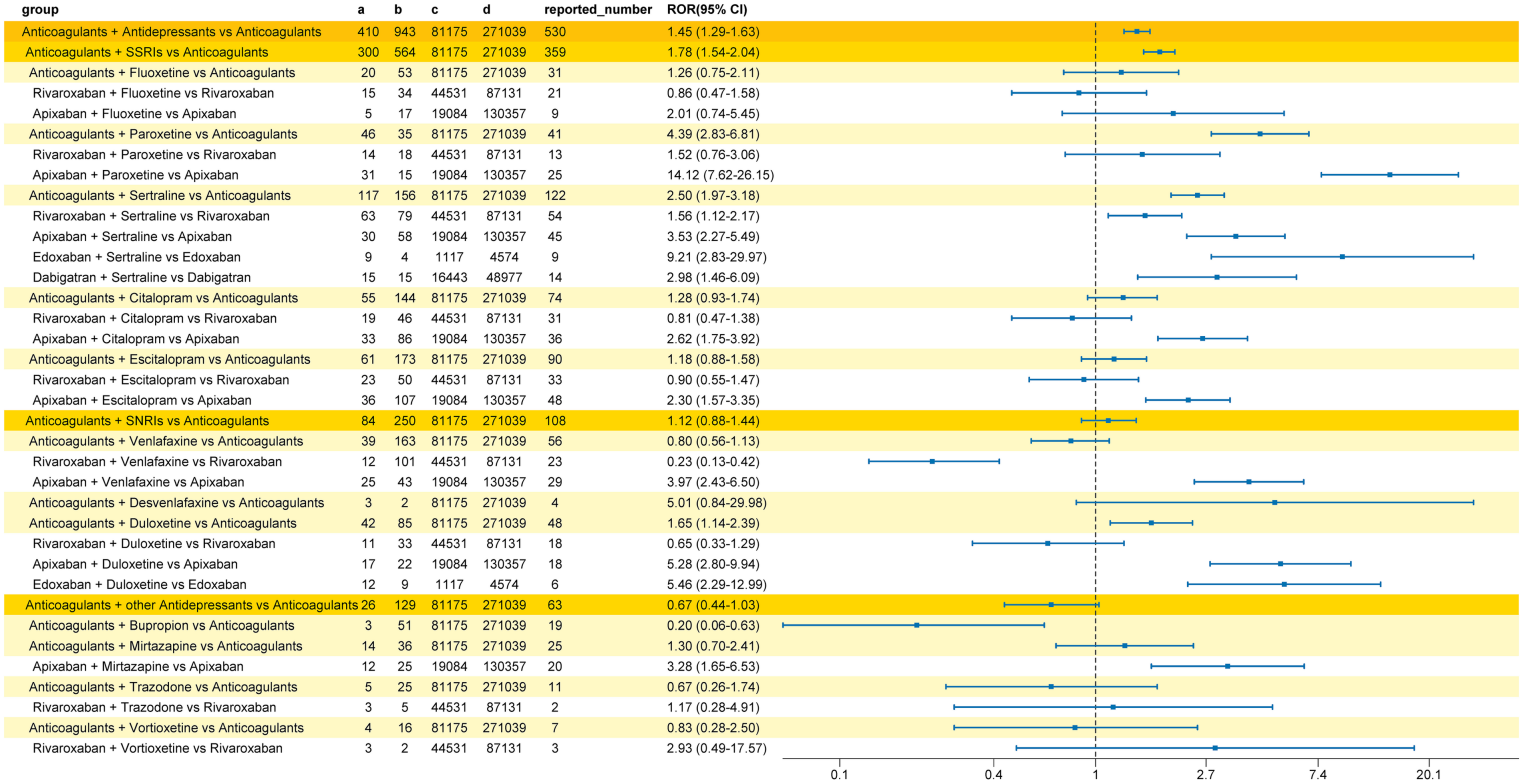
Reporting odds ratio (ROR) of bleeding signals for concomitant use of DOACs and antidepressants. Displays the calculated ROR values and 95% confidence intervals for various drug combinations to identify potential risks of overall hemorrhage.

Signal analysis results, presented in Figure 2, indicated a significantly increased bleeding signal in the group co-administered with DOACs and antidepressants compared to DOACs alone (ROR 1.45, 95%CI 1.29–1.63). Subgroup analysis based on the antidepressant mechanism of action showed a significant bleeding signal for the co-administration of DOACs and SSRIs (ROR 1.78, 95%CI 1.54–2.04), while no increased bleeding risk signal was detected for DOACs combined with SNRIs (ROR 1.12, 95%CI 0.88–1.44) or DOACs combined with other classes of antidepressants (ROR 0.67, 95%CI 0.44–1.03). Among SSRIs, paroxetine (ROR 4.39, 95%CI 2.83–6.81) and sertraline (ROR 2.50, 95%CI 1.97–3.18) were associated with an increased bleeding risk when combined with anticoagulants. Further subgroup analysis of DOACs revealed that co-administration of apixaban with paroxetine (ROR 14.12, 95%CI 7.62–26.15), rivaroxaban with sertraline (ROR 1.56, 95%CI 1.12–2.17), apixaban with sertraline (ROR 3.53, 95%CI 2.27–5.49), edoxaban with sertraline (ROR 9.21, 95%CI 2.83–29.97), dabigatran with sertraline (ROR 2.98, 95%CI 1.46–6.09), apixaban with citalopram (ROR 2.62, 95%CI 1.75–3.92), and apixaban with escitalopram (ROR 2.30, 95%CI 1.57–3.35) was significantly associated with a higher bleeding risk. Among SNRIs, the bleeding signal was significant for duloxetine co-administered with anticoagulants (ROR 1.65, 95%CI 1.14–2.39). Duloxetine increased the bleeding risk for apixaban (ROR 5.28, 95%CI 2.80–9.94) and edoxaban (ROR 5.46, 95%CI 2.29–12.99). No bleeding signal was detected for venlafaxine or desvenlafaxine co-administered with anticoagulants, though a significant signal was observed in the DOACs subgroup analysis for apixaban combined with venlafaxine (ROR 3.97, 95%CI 2.43–6.5). The strongest bleeding signal across all interaction groups was for the combination of apixaban and paroxetine (ROR 14.12, 95%CI 7.62–26.15). Among other classes of antidepressants, mirtazapine enhanced the bleeding risk of apixaban (ROR 3.28, 95%CI 1.65–6.53).

**Figure 3:**
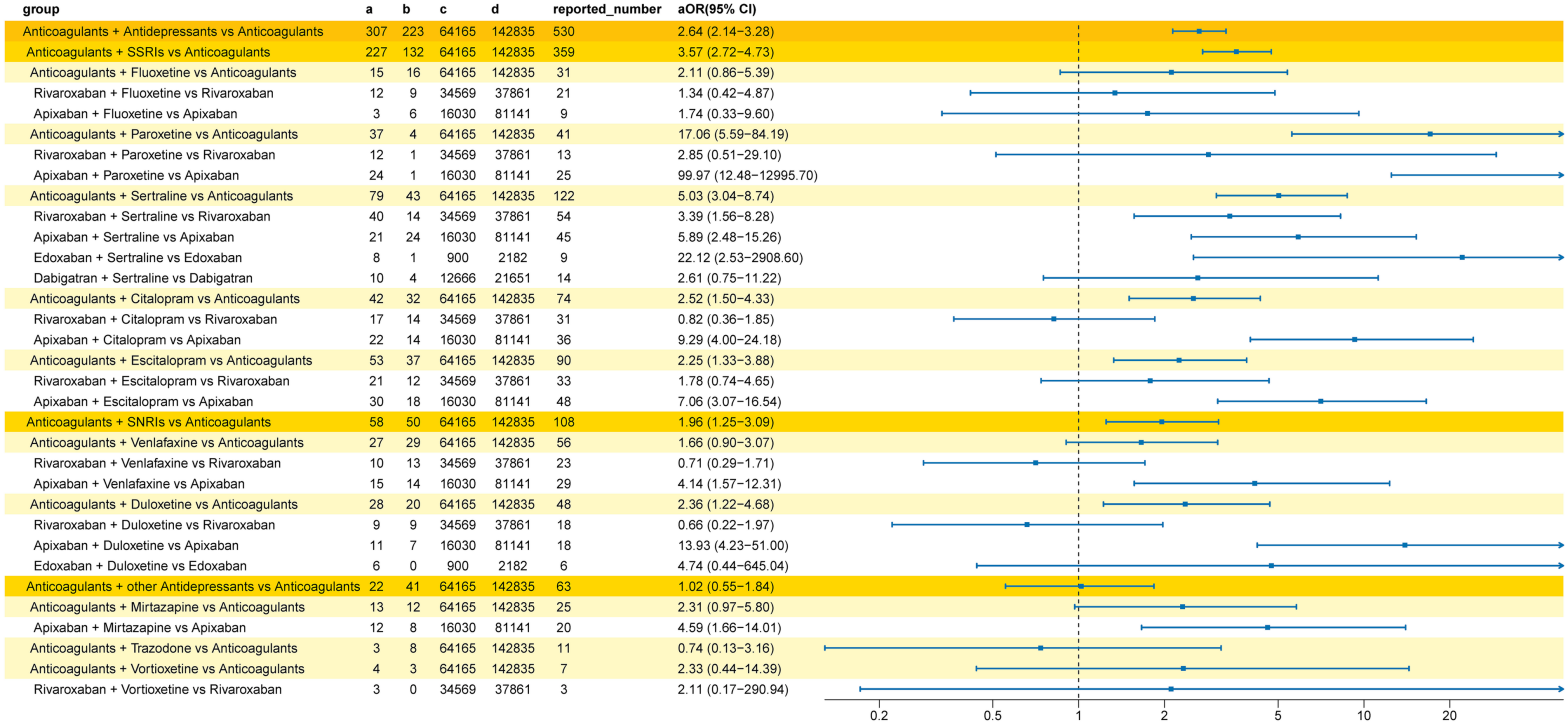
Adjusted reporting odds ratios (aOR) for the bleeding signal associated with the co-administration of DOACs and antidepressants. Presents findings from the multiple logistic regression analysis after adjusting for potential confounding factors like age and gender.

The results of the multiple Logistic regression are shown in Figure 3. After adjustment for potential confounding factors (including sex, reporter type, and age group), the bleeding signal detection results were largely consistent with the unadjusted analysis. The strongest bleeding signal remained in the apixaban and paroxetine co-administration group (aOR 99.97, 95%CI 12.48–12,995.70). Differences included newly detected bleeding signals for DOACs combined with SNRIs (aOR 1.96, 95%CI 1.25–3.09), DOACs combined with citalopram (aOR 2.52, 95%CI 1.50–4.33), and DOACs combined with escitalopram (aOR 2.25, 95%CI 1.33–3.88). Conversely, the bleeding signals for edoxaban combined with duloxetine and dabigatran combined with sertraline were no longer detected after adjusting for confounding factors.

### 3.3 Further analysis of nervous system hemorrhage

**Figure 4:**
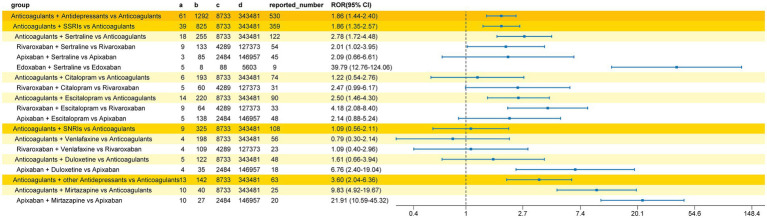
Reporting odds ratio (ROR) of nervous system bleeding signals for concomitant use of DOACs and antidepressants. Focuses specifically on the risk signals associated with neurological bleeding events for each drug pairing.

We conducted a subgroup analysis of nervous system bleeding events. In the interaction group (concomitant use of a DOAC and an antidepressant), we detected a total of 61 cases of nervous system hemorrhage. For comparison, in the group receiving DOACs alone, 8,201 cases of nervous system hemorrhage were identified. The specific Preferred Terms (PTs) for the 61 cases in the interaction group are detailed in Table 4. The analysis results (Figure 4) showed a significant nervous system hemorrhage signal for the combination of antidepressants and DOACs compared to DOACs alone (ROR 1.86, 95%CI 1.44–2.40). Among these, the strongest bleeding signal was observed in the group co-administered with other classes of antidepressants and DOACs (ROR 3.60, 95%CI 2.04–6.36). Mirtazapine was detected to increase the risk of nervous system bleeding with anticoagulants (ROR 9.83, 95%CI 4.92–19.67), with a significantly increased risk for the combination of mirtazapine and apixaban (ROR 21.91, 95%CI 10.59–45.32). The bleeding signal for SSRIs combined with DOACs was the second strongest (ROR 1.86, 95%CI 1.35–2.57), where sertraline (ROR 2.78, 95%CI 1.72–4.48) and escitalopram (ROR 2.50, 95%CI 1.46–4.30) were found to increase the bleeding risk of DOACs. Bleeding signals were detected for the combinations of sertraline with rivaroxaban (ROR 2.01, 95%CI 1.02–3.95), sertraline with edoxaban (ROR 39.79, 95%CI 12.76–124.06), and escitalopram with rivaroxaban (ROR 4.18, 95%CI 2.08–8.40). No increased risk of nervous system bleeding was detected for SNRIs combined with DOACs (ROR 1.09, 95%CI 0.56–2.11), but co-administration of duloxetine and edoxaban was associated with an increased risk of nervous system hemorrhage (ROR 6.76, 95%CI 2.40–19.04). Among all interaction groups, the combination of edoxaban and sertraline showed the strongest nervous system bleeding signal (ROR 39.79, 95%CI 12.76–124.06).

**Table 4:** Preferred terms (PTs) for nervous system hemorrhage.

PT	*n* (%)
Cerebral hemorrhage	23 (37.7%)
Haemorrhagic stroke	13 (21.3%)
Hemorrhage intracranial	10 (16.4%)
Cerebral haematoma	7 (11.5%)
Central nervous system hemorrhage	4 (6.56%)
Subarachnoid hemorrhage	2 (3.28%)
Cerebellar haematoma	2 (3.28%)

Lists the specific MedDRA-standardized terms used to identify the 61 cases of nervous system bleeding in the interaction group.

**Figure 5:**
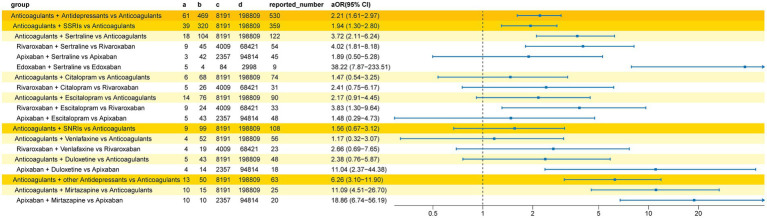
Adjusted reporting odds ratios (aOR) for the nervous system hemorrhage signal associated with the co-administration of DOACs and antidepressants. Shows the results of the multivariate analysis specifically targeting nervous system hemorrhage signals.

After adjusting for potential confounding factors (including sex, reporter type, and age group), the results from the multiple Logistic regression analysis (Figure 5) were largely consistent with the unadjusted analysis. The strongest bleeding signal remained in the edoxaban and sertraline co-administration group (aOR 38.22, 95%CI 7.87–233.51). Following adjustment, no bleeding signal was detected for the DOACs combined with escitalopram group (aOR 2.17, 95%CI 0.91–4.45).

## 4 Discussion

This study used real-world data from the FAERS database to explore the impact of antidepressants on DOAC-related bleeding risk. The results showed that compared with DOAC monotherapy, concurrent use of antidepressants significantly increased the bleeding risk, among which the combination of SSRIs and DOACs had the highest bleeding risk. Subgroup analysis of nervous system hemorrhage also indicated that the risk of the combined regimen was higher than that of DOAC monotherapy. This finding is consistent with the conclusions of multiple high-quality cohort studies and meta-analyses (9, 14, 16, 33). For example, a large cohort study in Stockholm, Sweden, found that the incidence of severe bleeding in the combined group (4.7 cases per 100 person-years) was significantly higher than that in the monotherapy group (2.7 cases per 100 person-years), with an adjusted Hazard Ratio (aHR) of 1.42 (9).

To minimize interference from other drug–drug interactions, we implemented rigorous inclusion and exclusion criteria, resulting in a relatively small sample size. Within our included cohort, 80.5% of the cases were individuals over 60 years of age. Several factors may account for this: first, cardiovascular diseases are prevalent among the elderly, making this population the primary recipients of anticoagulant therapy; furthermore, elderly patients with cardiovascular conditions are more likely to present with comorbid depression (6, 12, 34). Secondly, age-related declines in renal and hepatic functions, alongside changes in body composition, significantly alter the metabolism and clearance rates of therapeutic agents, which subsequently leads to an increased incidence of adverse drug reactions (35). This serves as a critical physiological basis for the elevated bleeding risk observed in elderly patients (36). Thirdly, a high proportion of the elderly population suffers from multiple chronic diseases, leading to polypharmacy (37, 38). Additionally, the risk of adverse outcomes increases accordingly in patients receiving multiple medications (39).

Between 2008 and 2012, dabigatran, rivaroxaban, and apixaban were successively launched in Europe and the United States (40–42). Edoxaban was approved in Japan in 2011 and in the United States in 2015 (43). At those times, DOACs were not yet approved in many countries or included in formularies, resulting in lower international post-marketing sales, which in turn delayed the documentation of relevant post-marketing data. Regarding the reporter types, compared with the DOAC monotherapy group, the interaction group had a higher proportion of physician reporters (34.5% vs. 27.5%) and a lower proportion of consumers (33.3% vs. 55.2%). This suggests the presence of reporting bias—physicians may be more inclined to report bleeding events when they are aware of potential drug–drug interactions; therefore, we adjusted for this factor in our subsequent analysis.

The core mechanism underlying the increased bleeding risk is a pharmacodynamic (PD) interaction, mainly resulting from the direct effect of antidepressants on the hemostatic process. Platelet aggregation relies on the 5-hydroxytryptamine (5-HT)-mediated signaling pathway. SSRIs and some SNRIs can deplete intraplatelet 5-HT reserves by inhibiting the serotonin transporter (5-HTT) on the platelet membrane, thereby impairing platelet aggregation function and prolonging bleeding time (17, 18, 21, 23). This antiplatelet effect, combined with the inhibition of coagulation factors by DOACs, forms a “double-hit” that significantly increases the probability of bleeding events (19, 44).

The intensity of bleeding risk is positively correlated with the drug’s binding affinity for 5-HTT (45, 46): paroxetine, sertraline, and fluoxetine have high affinity for 5-HTT; citalopram, escitalopram, etc., have moderate affinity; trazodone, bupropion, and mirtazapine have very low affinity. The lower the affinity, the weaker the inhibition of 5-HT reuptake (47). Shao et al. pointed out that in atrial fibrillation patients receiving oral anticoagulants, initiating SSRIs is associated with a higher bleeding risk than starting other types of antidepressants (8). In the elderly population, the absolute difference in upper gastrointestinal bleeding between users of high-affinity antidepressants and those of low-affinity antidepressants increases with age and the severity of underlying diseases (48). Consistent with this, our study found that the combination of SSRIs and DOACs had the highest bleeding risk (ROR 1.78, 95%CI 1.54–2.04), while no significant bleeding signals were detected for SNRIs and other classes of antidepressants (SNRIs: ROR 1.12, 95%CI 0.88–1.44; other classes: ROR 0.67, 95%CI 0.44–1.03).

Mirtazapine (a noradrenergic and specific serotonergic antidepressant) and bupropion (a norepinephrine-dopamine reuptake inhibitor) are often recommended as alternative medications for patients at high bleeding risk due to their lack of 5-HT reuptake inhibition (49, 50). However, a meta-analysis found no difference in bleeding risk between mirtazapine and SSRIs (51). A potential mechanism is mirtazapine’s strong antagonistic effect on the 5-HT2A receptor, as 5-HT-mediated enhancement of platelet activation in whole blood is mediated by this receptor (52, 53). Our study also confirmed that mirtazapine can increase the bleeding risk of apixaban, and the risk of nervous system hemorrhage associated with their combination is significantly increased, suggesting that it is premature to recommend mirtazapine as an alternative for patients at high bleeding risk.

Intracranial hemorrhage (ICH) is the most dangerous complication in atrial fibrillation patients treated with DOACs, with an annual incidence rate of 0.23–0.80% (54–57). DOAC-related ICH is more severe and has a higher mortality rate than spontaneous ICH (58). Currently, studies on ICH caused by the combination of DOACs and antidepressants are limited, mostly focusing on the combination of SSRIs and anticoagulants (10, 44, 59). Subgroup analysis of nervous system hemorrhage in this study found that SSRIs can increase the risk of DOAC-related cerebral hemorrhage, which is consistent with previously reported research results (60). Specific combinations with strong bleeding signals included edoxaban + sertraline, rivaroxaban + sertraline, and rivaroxaban + escitalopram. In addition, strong bleeding signals were observed for the combination of apixaban with mirtazapine/duloxetine. Therefore, we recommend maintaining high vigilance for nervous system hemorrhage associated with the combined use of anticoagulants and antidepressants (especially SSRIs).

This study has several limitations. First, as a spontaneous reporting system, the FAERS possesses inherent defects, including suboptimal data quality, missing values, and duplicate reports. A significant proportion of submissions by non-healthcare professionals may lead to data inconsistencies or misreporting, thereby introducing potential bias. Second, the data primarily originate from the United States, which may be influenced by factors such as ethnicity, geography, and variations in clinical practice, limiting the generalizability of the findings. Third, bleeding risk is dose-dependent; however, the lack of data on drug dosages and renal function makes it difficult to determine whether the observed risk arises from the drug class itself, individual patient factors, or medication misuse. Fourth, a limited number of reports for certain combinations may inflate signal scores in frequency-based statistical methods, potentially leading to instability in the results. Fifth, due to missing information or small case counts, this study did not adjust for key confounding factors such as DOAC indications (AF vs. VTE), renal function, bleeding history, alcohol consumption, or frailty status. Consequently, these findings should be interpreted with caution in real-world clinical applications.

## 5 Conclusion

This study revealed that the co-administration of antidepressants and DOACs significantly increases the risk of bleeding, particularly the risk of nervous system hemorrhage. Among the different classes of antidepressants, SSRIs combined with DOACs showed the highest bleeding risk. However, the bleeding risk associated with mirtazapine, which lacks 5 − HT reuptake inhibition, when combined with DOACs should also not be overlooked. This underscores the need for clinicians to cautiously evaluate the bleeding risk during co-medication. Future research should further explore the underlying mechanisms and optimize therapeutic strategies.

## Data Availability

The original contributions presented in the study are included in the article/supplementary material, further inquiries can be directed to the corresponding author.

## Footnotes

1
https://fis.fda.gov/extensions/FPD-QDE-FAERS/FPD-QDE-FAERS.html
